# Seroepidemiology (2018–2024) and epidemic spread of an emerging human parvovirus B19 genotype 1 (subtype 1a2) variant in Hungary, 2023/2024

**DOI:** 10.1007/s00705-025-06228-2

**Published:** 2025-02-02

**Authors:** Fruzsina Tóth, Tímea Moser, Ákos Boros, Benigna Balázs, Károly Takáts, Péter Pankovics, Gábor Reuter

**Affiliations:** Department of Medical Microbiology and Immunology, Medical School, University of Pécs, Szigeti út 12., Pécs, 7624 Hungary

## Abstract

An unusually large number of human parvovirus B19 (B19V) infections were reported in European countries in 2023/2024, but the genetic background of this B19V epidemic strain is unknown. In this study, there was a larger number of confirmed B19V infections (five in 2021, eight in 2023, and 59 in 2024) and higher IgG seroprevalence (41.4% in 2022 and 54.3% in 2024) in Transdanubia, Hungary, in 2024 compared to 2018-2023. A B19V genotype 1a2 variant (prototype, 1338/HUN/2024, PQ155933) with common and unique nucleotide insertions in the untranslated regions of the genome and nonsynonymous and synonymous mutations in the coding region (NS1 and VP1) could be responsible to the ongoing B19V epidemic in Europe.

**Supplementary Information:**

The online version contains supplementary material available at 10.1007/s00705-025-06228-2.

Human parvovirus B19 (B19V) is a well-known human pathogen belonging to the genus *Erythroparvovirus*, family *Parvoviridae* [1]. B19V is a small, non-enveloped virus with a linear, single-stranded DNA genome of ~5.6 kb with identical inverted terminal repeat sequences at each end. The genome contains two open reading frames (ORFs) encoding a non-structural protein (NS1 or Rep) and two structural proteins (VP1 and VP2). Three B19V genotypes (gt1, gt2, and gt3) and four subtypes (gt1a, gt1b, gt3a, and gt3b) have been identified [2], with gt1 being the most prevalent [3–5]. Subtype gt1a is further divided into gt1a1 and gt1a2, which differ mostly due to synonymous mutations distributed throughout the genome [6].

B19V can be transmitted by respiratory secretions, hand-to-mouth contact, blood transfusion, or transplacental transmission. B19V infection is associated with a self-limiting febrile erythematous illness (called erythema infectiosum, fifth disease, or slapped cheek disease), mainly in children, but asymptomatic infection and disease complications including anaemia, pancytopenia in immunocompromised patients, transient aplastic crisis in patients with chronic haematological disorders, arthritis, arthralgia, myocarditis, hepatitis, vasculitis, and central nervous system infections (encephalitis, meningitis, peripheral neuropathy) have been documented [7]. Hydrops fetalis may develop in pregnancy by transplacental infection [8].A long-term study showed that B19V infections have a 1-year-long seasonal variation (with a peak in spring in Europe) and a 3- to 6-year-long periodicity of increased epidemic circulation superimposed on the annual epidemics [9]. B19V infection presently is not a reportable disease in most countries, and it is generally believed to cause only mild symptoms in most cases. Except in severe cases, most B19V infections are diagnosed clinically. Routine clinical laboratory diagnosis of B19V infections is predominantly based on serological tests and less often by molecular methods. Therefore, relatively few B19V genome sequences are available for molecular epidemiological purposes, and they are rarely analyzed in detail [2, 3, 10]. Preliminary data suggested an unusually large number of B19V infections in several European countries in the season 2023/2024 [11–14], but the genotypes and sequence characteristics of the circulating viruses associated with this epidemic are unknown.

There are only limited data available about B19V infections in Hungary. Two case studies with disease complications [15, 16] and a summary of clinical symptoms in a case series from 2011 in Hungarian [17] are listed in the PubMed database, but there is no information available about the seroepidemiology and circulating genotypes of B19V in the country.

In this study, we investigated the seroepidemiology and epidemic spread of B19V and characterized the complete protein coding region of emerging genotype 1a (subtype 1a2) B19V variants in Hungary that are likely to be responsible for the ongoing epidemic in Europe.

The seroepidemiological analysis was based on a retrospective analysis of the serological laboratory results of B19V immunoglobulin (Ig)G and IgM antibody tests performed between January 1, 2018, and July 31, 2024. A total of 1441 blood samples from patients of all ages were originally sent by physicians (university/county hospitals and general practitioners) for diagnostic purposes to the Laboratory of Virology, Department of Medical Microbiology and Immunology, University of Pécs (Pécs, Hungary), which covers a total population of 864,299 inhabitants (8.92% of the total population of Hungary in 2022) in three counties (Baranya, Somogy, and Tolna) in the South Transdanubia region of southwestern Hungary. South Transdanubia is one of the seven regions of Hungary. The study protocol conformed to the ethical guidelines of the 1975 Declaration of Helsinki. The health data collection authorization number for this study is KK/3937-1/2024 (University of Pécs).

Serum samples were tested by the ELISA method using parvovirus B19 IgG (Dia.Pro Diagnostic Bioprobes, Sesto San Giovanni, Italy) and parvovirus B19 IgM (Dia.Pro Diagnostic Bioprobes, Sesto San Giovanni, Italy) test kits according to the manufacturer's instructions. These assays allow the qualitative detection of anti-parvovirus B19 IgG and IgM antibodies in human serum and plasma samples (https://www.diapro.it/). According to the manufacturer's validation criteria, the sample is positive for B19V IgG and IgM antibodies if the optical density (OD) is equal to or higher than 1.20 compared to the OD/cutoff ratio. A positive test result for B19V IgM may indicate an acute (recent) B19V infection, while a positive result for B19V IgG antibody may indicate a previous infection.

Selected serum samples that were positive for parvovirus B19 IgM antibodies were tested for parvovirus B19 DNA by conventional PCR and Sanger sequencing, using an AB3500 Genetic Analyzer (Applied Biosystems, Hitachi, Tokyo, Japan). Primer pairs for nested PCR were designed based on the prototypes of the B19V genotypes (gt1-3) and subtypes (Fig. 3) and used for B19V screening and characterization of the continuous ~4550-nucleotide-long complete protein coding genome region. The primer sequences are shown in Supplementary Table S1.

The selection of the 5-year age groups was made using the MedBakter program (Prolab Kft., Hungary). Data were analyzed and figures were prepared using Microsoft Excel 2013 (Redmon, WA, US).

Between January 1, 2018, and July 31, 2024, a total of 1,441 specimens were tested for B19V IgG and IgM antibodies (Fig. 1). The yearly distribution of the samples had an increasing trend; however, there was a decline in 2020 within the SARS-CoV-2 pandemic period. Over the study period, the seropositivity of the B19V IgG antibody varied between 41.4% (in 2022) and 54.3% (in 2024). Throughout the study period, the overall seroprevalence was 48.3%. Five B19V IgM-positive samples were identified in 2021, eight in 2023, and 59 in 2024 (Fig. 1). The average age of the patients with B19V-IgM-positive specimens was 29.96 years (range: 2 weeks-85 years), and 73.6% of these patients were female.

**Fig. 1:**
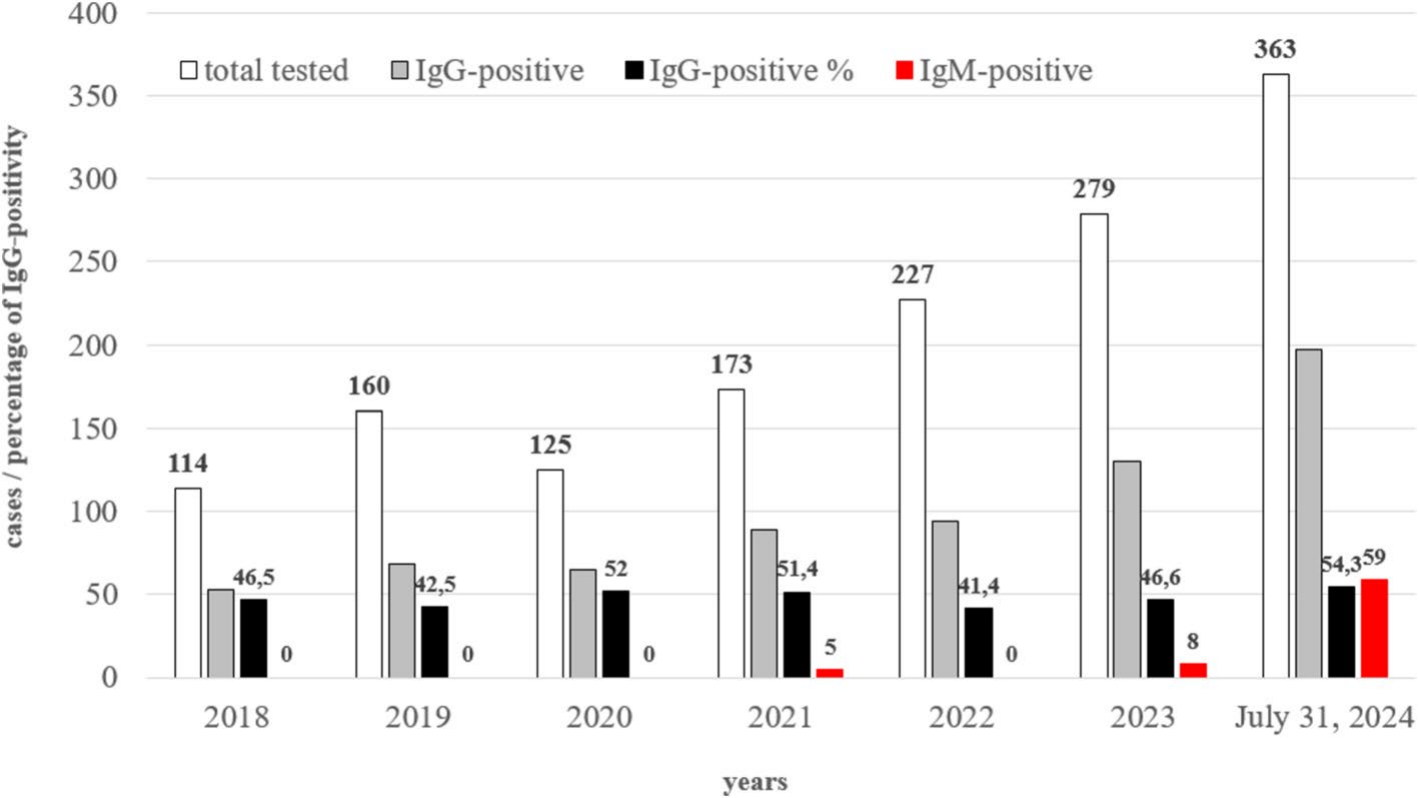
Yearly distribution of serum samples (N = 1441) tested for parvovirus B19 (B19V) IgG and IgM antibodies in South Transdanubia, Hungary, between 2018 and 2024. White and grey columns represent the total numbers of the tested and B19V IgG-positive specimens, respectively. The black column represents the percentage (%) of B19V IgG seropositivity per year. The red column represents the number of B19V IgM-positive samples (N = 72).

When comparing the cumulative number of B19V-IgG-seropositive individuals by age group between 2018 and 2023 and the data from 2024, a different pattern of was observed (Fig. 2). In 2024, a higher B19V IgG seropositivity rate (%) was found in the age groups 6-10 years (3.16 times), 11-15 years (1.79 times), 16-20 years (1.39 times), and 21-25 years (1.16 times) than in the cumulative data in 2018-2023. Between 2018 and 2023, 36.8-60.5% of the population in the age groups from 21-25 years to 36-40 years were seropositive. These groups included pregnant women. Seropositivity was 70% in the age group of 41-45 years. In the age groups from 46-50 years to 91-95 years, the seropositivity ranged from 47.6% to 80%.

**Fig. 2:**
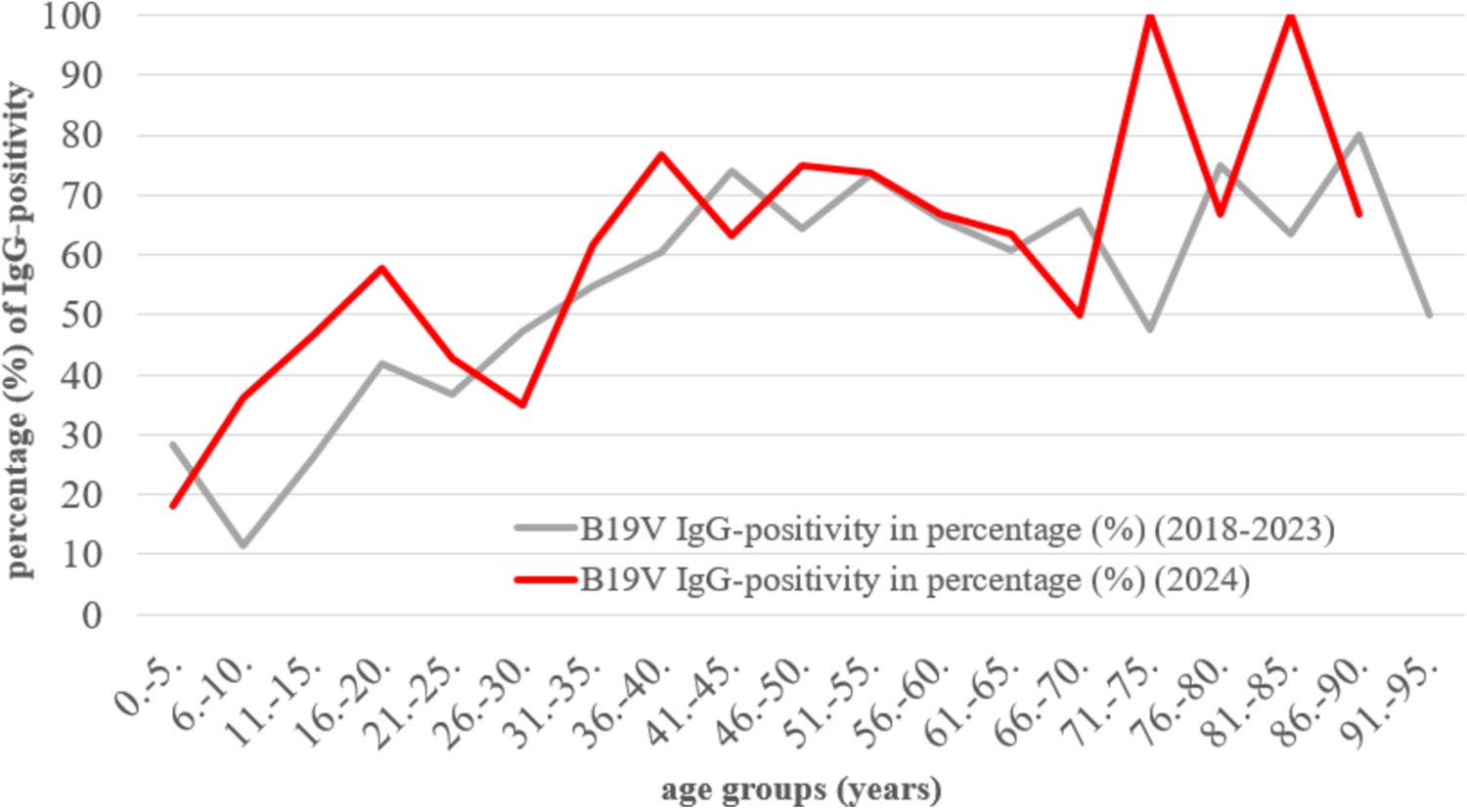
Percentage (%) of parvovirus B19 (B19V) IgG antibody positivity by age groups between 2018 and 2023 (N = 1078; cumulative data in grey) and in 2024 (N = 363; red). Each age group covers 5 years from 0 to 95 years. The sample size was less than 30 in the age groups between 81 and 95. Therefore, these data should be viewed with caution.

A total of 68 (94.4%) of the 72 B19V IgM-positive specimens were available for molecular testing (only one of the samples from 2021 was available). A total of 51 (75%) of the 68 B19V IgM-positive specimens were positive for B19V DNA by conventional nested PCR using screening primers (Supplementary Table S1). The comparative dataset of the serological (x = IgM OD/cutoff and y = IgG OD/cutoff) and nested PCR (first and second round) test results of the B19V IgM-positive samples are shown in Supplementary Figure S1. The first- and second-round PCR-positive samples were grouped among IgM-positive/IgG-negative and high IgM-positive/IgG-positive samples (Supplementary Fig. S1). Ten B19V-IgM-positive/PCR-positive specimens were randomly selected (three from 2023 and seven from 2024) for sequencing of the complete coding region (NS1 and VP1) of the genome (Supplementary Fig. S1). Phylogenetic analysis showed that all 10 of these isolates belonged to gt1 subtype gt1a2, but they were divided into two sequence groups (Fig. 3). Nine B19V strains (PQ155930-PQ155938) clustered together (with less than 0.3% nt sequence divergence in the ~4,550-nt-long genome sequence), but B19V strain 3554/HUN/2024 (GenBank PQ155939) formed a distinct lineage (Fig. 3). Strain 1338/HUN/2024, the prototype strain of this cluster, showed 99.89% and 99.80% nt sequence identity to the closest B19V strains, which were from Slovakia (GenBank no. OZ031174) in 2024 and France (GenBank no. OR138122) in 2019, respectively. The 1338/HUN/2024-like B19V strains (Fig. 3) have both common and unique mutations in the 5’UTR (insertion of G at nt position 386/387 relative to the reference strain M13178), nonsynonymous (Cys→Ser at aa position 17, Gly→Ala at aa 159, and Thr→Asn at aa 163) and synonymous (T→C at nt position 942, C→T at nt 1405, and A→G at nt 1524) mutations in NS1 and nonsynonymous (Ala→Thr at aa 260 in the antigenic determinant region) and synonymous (G→A at nt 2500, A→G at nt 2527, G→A at nt 3221, T→A at nt 3517, A→C at nt 3571,and A→C at nt 3,658) mutations in VP1 that had not been detected previously in B19V strains.

**Fig. 3:**
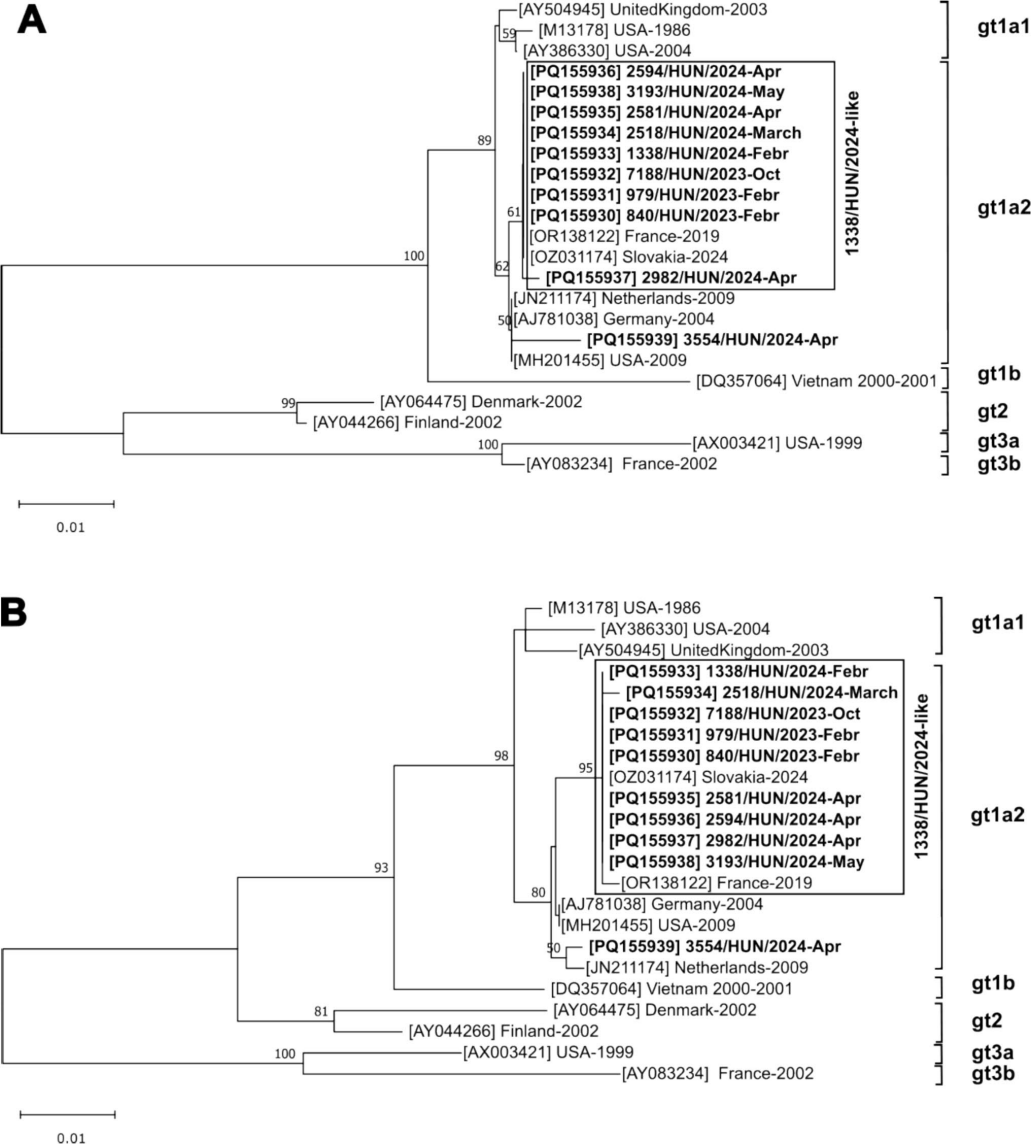
Phylogenetic analysis based on NS1 (A) and VP1 (B) nucleotide (nt) sequences of human parvovirus B19 (B19V) strains from this study (N = 10, indicated by bold letters), the most similar sequences identified by BLASTn search, and other representative genotypes/subtypes of B19V. The 1338/HUN/2024-like epidemic B19V strains are shown in a frame. Neighbor-joining trees were generated based on nucleotide sequence alignments of the complete NS1 (2016 nt) and VP1 (2346 nt) regions, using the Tamura-3+G model in MEGA ver. 11 with 1000 bootstrap replicates. Only bootstrap values higher than 50 are shown. The scale bars indicate the number of nucleotide substitutions per site. The designation of the sequences is as follows: accession number in square brackets, followed by country of origin and collection date. gt, genotype

This study provides new information about the seroepidemiology of B19V and the epidemic spread of an emerging B19V variant in South Transdanubia, Hungary, in 2023/2024. The first genetically confirmed cases of the epidemic were identified in February 2023, but the outbreak expanded in winter 2023/spring 2024. During the time of our investigation at the end of 2023, an unusually large number of B19V infections and illnesses were reported in Hungary (personal information, media news, unpublished data) and other European countries [11–14].

Sequence comparisons of the complete coding region of the genome revealed that highly similar genotype 1 (subtypes 1a2) viruses were circulating throughout 2023 and 2024 (the prototype strain is HUN1338/2024, PQ155933) that were genetically very similar to B19V viruses found in Slovakia in 2024 and France in 2019. Other B19V viruses with sequences in the GenBank database were found to be more distantly related genetically (and temporally) to our prototype strain. We therefore hypothesize that the HUN1338/2024-like subtype 1a2 B19V strain was the predominant circulating epidemic strain in the population in Hungary, and probably in other parts of Europe (and maybe Israel), in 2023 and 2024 [10, 12, 13, 18]. Further multi- or international comparative studies are needed to determine the actual genotype distribution and confirm the identification of the present epidemic B19V strain in Europe.

In addition to an increased rate of B19V IgM and PCR positivity, an increase in B19V seroprevalence was found in the age groups between 6 and 25 years in South Transdanubia in 2024. Although we did not analyze the transmission modes of B19V in detail, a medical history of person-to-person (e.g., between siblings with hereditary spherocytosis and transient aplastic crisis; 1338/HUN/2024), transfusion-associated (e.g., 2518/HUN/2024), and transplacental transmission was also identified in clinical cases in connection with the microbiological diagnostic work. These data also suggest a massive spread of B19V in the study population over a one-year period.

Several natural and human factors (e.g., the ratio of the immunological naïve population, annual cycles, a 3- to 6-year-long periodicity, differences in reporting systems, screening strategies for blood testing, etc.) are known to influence reported frequency of the B19V epidemic infections [10, 19–21] and might have played a substantial role in 2023/2024 in Hungary and other countries. The last documented B19V epidemic peak in Europe was in 2016/2017 [9, 14], and the average length of the interepidemic period for B19V was 4 years. Thus, the next epidemic would have been expected around 2020/2021, which coincided with the SARS-CoV-2 pandemic in Europe. The transmission dynamics of B19V could therefore have been affected by SARS-CoV-2 restrictions and containment measures, and this might be why the B19V interpandemic time period was prolonged and nearly doubled. This might also have played a significant role in the increased number of infections and cases of disease among the accumulated immunologically naïve populations, as hypothesized recently [9, 22], and also – based on the similar atypical disease patterns seen – in other (viral) infections [23, 24]. In addition to the epidemiological description, the dynamics and the molecular epidemiology of the B19V epidemic can be correctly interpreted by knowing the genetic properties of the pathogen. In the genome of the prototype B19V study strain (1338/HUN/2024) and its relatives, we found a total of four synonymous (3 in NS1 and 1 in VP1) and nine nonsynonymous (both common and unique) genome mutations in the coding region, as well as a nucleotide insertion in the 5’UTR. The description of genetic changes over time – as seen in newly emerged SARS-CoV-2 variants [25, 26] – can lead to important new knowledge, but currently, the significance of the mutations in B19V for disease severity, transmissibility, receptor affinity, and disease epidemiology is not known [27, 28]. A more accurate basis for studying the epidemiology, number of infections, and dynamics of B19V infections could be obtained if reporting of clinical and laboratory-confirmed cases were compulsory in each country and if we had more molecular epidemiological data with complete B19V genome sequence information.

## Supplementary Information

Below is the link to the electronic supplementary material.

Supplementary file1 (DOCX 218 KB)

Supplementary file2 (DOCX 15 KB)

## Data Availability

The nucleotide sequence data reported here are available in the DDBJ/EMBL/GenBank databases under the accession numbers PQ155930-PQ155939.
