# Supplementary material for: Seroepidemiology (2018–2024) and epidemic spread of an emerging human parvovirus B19 genotype 1 (subtype 1a2) variant in Hungary, 2023/2024
Source: Arch Virol. 2025 Feb 2;170(2):45. doi: 10.1007/s00705-025-06228-2 (PMC11788230; doi:10.1007/s00705-025-06228-2)

**Figure S1.** Distribution of the B19V ELISA IgM (x-axis) and IgG (y-axis) OD/cut-off scores of the B19V IgM-positive serum samples (N=72), in addition to the nested-PCR (first and second round) test results (N=68). The IgM-positive and IgG-positive ELISA threshold limits are OD/cut-off > 1.20 (dashed line). The B19V IgM-positive/PCR-negative samples are marked by a black circle (N=17), the IgM-positive/only second round nested PCR-positive specimens are marked by green circle (N=37), the IgM-positive/first and second round nested PCR-positive specimens are marked by red circle (N=14) and the IgM-positive/not available specimens for nested PCR are marked by blue circle (N=4). Specimens selected for DNA sequencing (N=10) for complete coding genome region characterization are marked by **bold** letters and purple colour. The designation of the specimens is the following: specimen code followed by collection date (year).


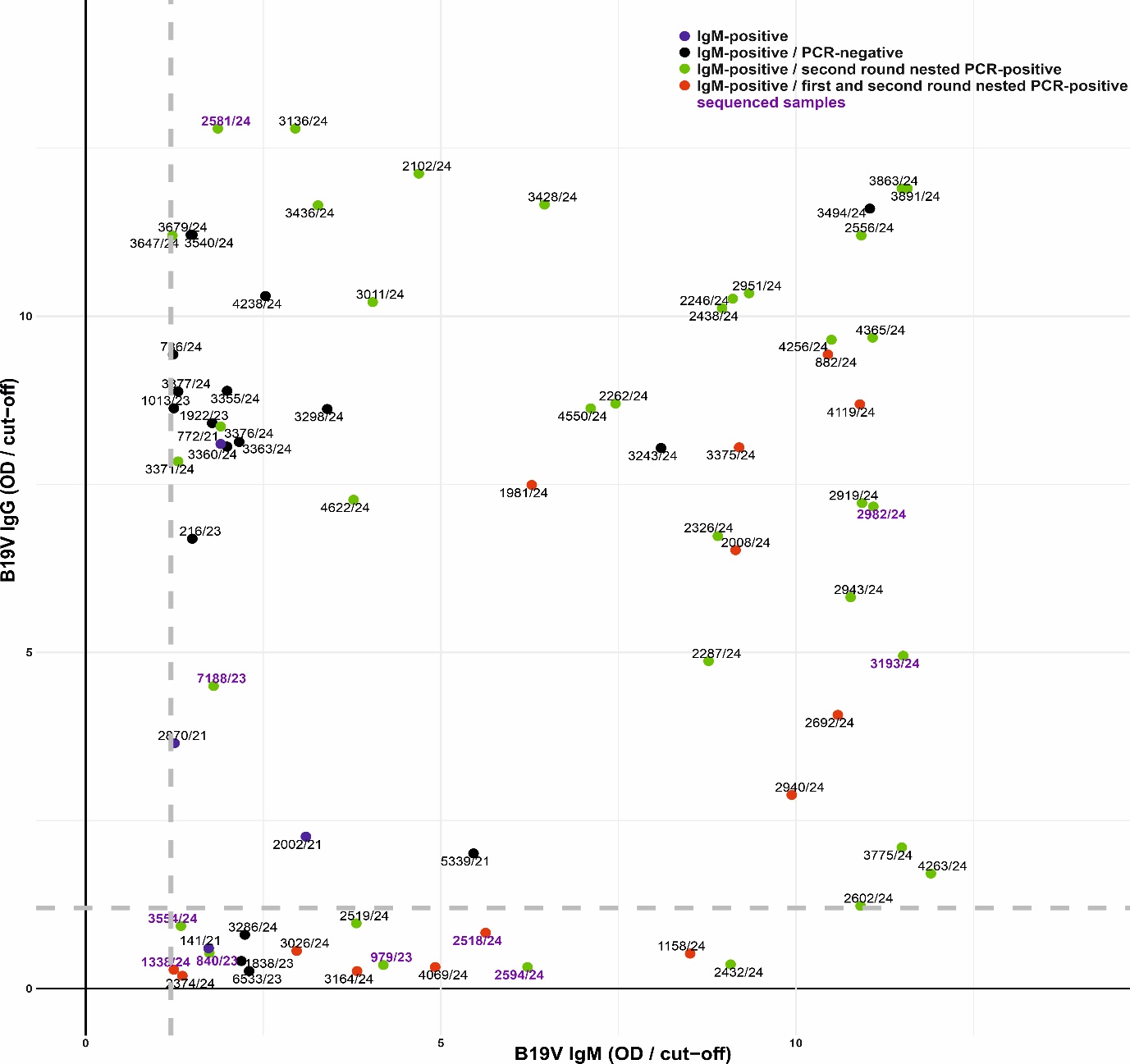

Supplement: Supplementary file 1 — Supplementary file1 (DOCX 218 KB) [file 705_2025_6228_MOESM1_ESM.docx]
