# Supplementary material for: Seroepidemiology (2018–2024) and epidemic spread of an emerging human parvovirus B19 genotype 1 (subtype 1a2) variant in Hungary, 2023/2024
Source: Arch Virol. 2025 Feb 2;170(2):45. doi: 10.1007/s00705-025-06228-2 (PMC11788230; doi:10.1007/s00705-025-06228-2)
Supplement: Supplementary file 2 — Supplementary file2 (DOCX 15 KB) [file 705_2025_6228_MOESM2_ESM.docx]

**Table S1:** List of B19V oligonucleotide primers used in this study which are suitable for determining the continuous partial 5’UTR and the complete protein-coding genome region (~4,550nt). VP1: viral capsid protein 1, NS1: non-structural protein-encoding genome regions, 5’UTR: untranslated region. *Numbers in the names of these primers used for NS1 and VP1 typing refer to the first or last positions of binding sites of the forward or reverse primers, respectively, on reference complete genome of human parvovirus B19 (NC_000883.2). nPCR: nested PCR.

| **Target region** | **Primer name** | **5’-3’ sequence** | **Reaction type** | **Product length (bp)** | **Reference**  **(Acc. no.)** |
| --- | --- | --- | --- | --- | --- |
| NS1-VP1 | ParvoB19-e1855-F1 | CACTATGAAAACTGGGCAA | Screening nPCR 1st round | 1239 | Servant et al., 2002 |
| NS1-VP1 | ParvoB19-3094-R1 | GGGAACTTCCGGCAAACTTCCTTG | Screening nPCR 1st round |  | Servant et al., 2002 |
| NS1-VP1 | ParvoB19-e1863-F2 | AAACTGGGCAATAAACTACAC | Screening nPCR 2nd round | 1168 | Hübschen et al., 2009 |
| NS1-VP1 | ParvoB19-3031-R2 | GTAGTCTTTTACTACTTGTGCTTG | Screening nPCR 2nd round |  | Hübschen et al., 2009 |
| VP1 | B19-2962-F* | AGACTTACACAAGCCTGGGCAA | VP1 typing nPCR 1st round | 2007 | this study |
| VP1 | B19-4969-R* | TTACAATGGGTGCACACGGCT | VP1 typing nPCR 1st round |  | this study |
| VP1 | B19-3062-F* | GACAGTGCTGCAAGGATTCAT | VP1 typing nPCR 2nd round | 1879 | this study |
| VP1 | B19-4941-R* | GTCCACAATTCTTCAGGCTTTT | VP1 typing nPCR 2nd round |  | this study |
| 5’UTR-NS1 | B19-286-F* | TGACGTCACAGGAAATGACGTA | NS1 typing nPCR 1st round | 1968 | this study |
| 5’UTR-NS1 | B19-2254-R* | GYGTACTAGAGCGCGGGGT | NS1 typing nPCR first round |  | this study |
| 5’UTR-NS1 | B19-355-F* | GCGGCATCTGATTTGGTGTCTT | NS1 typing nPCR 2nd round | 1821 | this study |
| 5’UTR-NS1 | B19-2176-R* | CAGAGCTTTCACCACTGCT | NS1 typing nPCR second round |  | this study |
